# Presenilin 2-Dependent Maintenance of Mitochondrial Oxidative Capacity and Morphology

**DOI:** 10.3389/fphys.2017.00796

**Published:** 2017-10-12

**Authors:** Sabrina Contino, Paolo E. Porporato, Matthew Bird, Claudia Marinangeli, Rémi Opsomer, Pierre Sonveaux, Françoise Bontemps, Ilse Dewachter, Jean-Noël Octave, Luc Bertrand, Serena Stanga, Pascal Kienlen-Campard

**Affiliations:** ^1^Alzheimer Research Group, Institute of Neuroscience, Université catholique de Louvain, Brussels, Belgium; ^2^Pharmacology and Therapeutics, Institute of Experimental and Clinical Research, Université catholique de Louvain, Brussels, Belgium; ^3^Metabolic Research Group, de Duve Institute, Université catholique de Louvain, Brussels, Belgium; ^4^Pole of Cardiovascular Research, Institute of Experimental and clinical Research, Université catholique de Louvain, Brussels, Belgium

**Keywords:** presenilin, mitochondria, glycolysis, oxidative phosphorylation, cellular bioenergetics, Alzheimer's disease

## Abstract

Mitochondrial dysfunction plays a pivotal role in the progression of Alzheimer's disease (AD), and yet the mechanisms underlying the impairment of mitochondrial function in AD remain elusive. Recent evidence suggested a role for Presenilins (PS1 or PS2) in mitochondrial function. Mutations of PSs, the catalytic subunits of the γ-secretase complex, are responsible for the majority of inherited AD cases (FAD). PSs were shown to be present in mitochondria and particularly enriched in mitochondria-associated membranes (MAM), where PS2 is involved in the calcium shuttling between mitochondria and the endoplasmic reticulum (ER). We investigated the precise contribution of PS1 and PS2 to the bioenergetics of the cell and to mitochondrial morphology in cell lines derived from wild type (PS+/+), PS1/2 double knock-out (PSdKO), PS2KO and PS1KO embryos. Our results showed a significant impairment in the respiratory capacity of PSdKO and PS2KO cells with reduction of basal oxygen consumption, oxygen utilization dedicated to ATP production and spare respiratory capacity. In line with these functional defects, we found a decrease in the expression of subunits responsible for mitochondrial oxidative phosphorylation (OXPHOS) associated with an altered morphology of the mitochondrial cristae. This OXPHOS disruption was accompanied by a reduction of the NAD^+^/NADH ratio. Still, neither ADP/ATP ratio nor mitochondrial membrane potential (ΔΨ) were affected, suggesting the existence of a compensatory mechanism for energetic balance. We observed indeed an increase in glycolytic flux in PSdKO and PS2KO cells. All these effects were truly dependent on PS2 since its stable re-expression in a PS2KO background led to a complete restoration of the parameters impaired in the absence of PS2. Our data clearly demonstrate here the crucial role of PS2 in mitochondrial function and cellular bioenergetics, pointing toward its peculiar role in the formation and integrity of the electron transport chain.

## Introduction

Metabolic dysfunction is central in Alzheimer's disease (AD) since it appears at very early stage of the disorder, even before clinical symptoms (Chen and Zhong, 2013). It is evidenced in patients by a decrease in glucose utilization in temporoparietal association areas, together with cognitive decline and a severe failure in mitochondria oxidative metabolism (Herholz, 2012). Mitochondria are known as the powerhouse of the cell for their capacity to supply energy, but they are also critical in other cellular processes such as apoptosis, reactive oxygen species (ROS) production (Paradies et al., 2014), and calcium homeostasis (Osellame et al., 2012). All these processes turn out to be affected in AD pathology (Hroudova et al., 2014). Studies carried out either on AD patients' tissue samples or AD transgenic mice models reported an array of mitochondrial dysfunctions, including a morphological shift toward fission (Wang et al., 2009), a disrupted motility (Wang et al., 2009; Xie et al., 2013), an impairment of the electron transport chain (ETC) (Bosetti et al., 2002), and an increase in ROS production with deleterious effects on mitochondrial DNA integrity (Onyango et al., 2006). In cytoplasmic hybrid (cybrid) cell lines, generated by insertion of platelets' mitochondria collected from sporadic AD (SAD) patients into human neuroblastoma cells (SHSY5Y) depleted of mitochondria, bioenergetics dysfunctions such as oxidative phosphorylation (OXPHOS) and glucose utilization defects have been observed (Silva et al., 2013). These metabolic perturbations found in AD raised a chicken and the egg issue, namely to define whether mitochondrial dysfunction is a cause or a consequence in this pathology.

Recent studies suggested that Presenilins are involved in the control of mitochondrial functions (Behbahani et al., 2006; Filadi et al., 2016). PS1 and PS2 are two homologous polytopic aspartyl proteases identified as the catalytic subunits of the γ-secretase complex. PSs are directly involved in AD since the production of Aβ is generated after the sequential cleavage of the Amyloid Precursor Protein (APP) by the β and the γ-secretase (Zheng and Koo, 2011). Aβ is the major component of senile plaques found in the brain of AD patients (Miller et al., 1993). Beside their key role in γ-secretase activity and Aβ production, mounting evidence indicate additional roles for PSs in cell physiology, for which their catalytic activity seems less evident. PSs are involved in calcium homeostasis (Zhang et al., 2013), autophagy (Lee et al., 2010), neurotransmitters release (Zhang et al., 2009), synaptic plasticity and memory (Saura et al., 2004). Moreover, although PSs have different localization in the cell with PS1 mainly located at the plasma membrane and PS2 in the trans-Golgi network and endosomal compartments (Meckler and Checler, 2016; Sannerud et al., 2016), they are both enriched in the mitochondria-associated membranes (MAM) (Area-Gomez et al., 2009). MAM are specific membrane domains connecting endoplasmic reticulum (ER) and mitochondria. They are involved in lipid metabolism, calcium and cholesterol homeostasis (van Vliet et al., 2014; Filadi et al., 2017). PS2 has been reported to regulate the formation of ER/mitochondria contacts (Filadi et al., 2016) and calcium cross-talk between these two organelles (Zampese et al., 2011a). It is hence of particular interest to define (i) the precise contribution of PSs to the multiple aspects of mitochondrial function and (ii) to understand the respective contribution of PS1 or PS2 to these processes. Unraveling the role of PSs in cell metabolism is crucial to get insight in their physiological function and to understand how PS gain- or loss-of-function can create a pathological context, related for instance to AD. We performed an array of metabolic measurements on Mouse Embryonic Fibroblasts cell lines (MEFs) derived either from wild type (PS+/+), PS1/2 double knock-out (PSdKO), PS2KO, and PS1KO mice embryos. We found a key role for PS2 (and not PS1) in cell metabolism and especially in OXPHOS and glycolysis. Indeed, the absence of PS2 altered the OXPHOS capacity and integrity but increased glycolytic flux to support energy needs. These pathways are crucial for energy-intensive consumer cells, like brain cells. Such defects might set the basis for further investigation of the metabolic impairments observed in AD.

## Materials and methods

### Cell lines and cell culture

Mouse Embryonic Fibroblasts cell lines (MEFs) derived from wild type (PS+/+), PS1/2 double knock-out (PSdKO), PS2KO and PS1KO mice embryos were previously described (Hebert et al., 2006; Marinangeli et al., 2015). Rescued cell lines refer here to MEFs stably re-expressing human PS1 or PS2 in the corresponding single KO background: PS2KO rescued by human PS2 (2R2) and PS1KO rescued by human PS1 (1R1). Cells were maintained in DMEM low glucose (5.5 mM) (Sigma-Aldrich, St Louis, USA) supplemented with penicillin/streptomycin solution (10 units–10 μg) and 10% fetal bovine serum (FBS) (Thermo Scientific, Rockford, USA). MEF 1R1 and 2R2 cell lines were generated by subcloning the human PSs cDNA sequences in the lentiviral backbone vectors plentiCMV/TOpuro and pTMtm898neo, respectively. Lentiviruses were produced in human embryonic kidney 293T cells. Supernatants containing the lentiviruses were concentrated with the Lenti-X Concentrator kit according to the manufacturer's instructions (Clontech Laboratories; California, USA). MEFs PS1KO and PS2KO were transduced by lentiviral particles followed by puromycin (2.5 μg/ml) selection (1R1) and G418 (500 μg/ml) selection (2R2), respectively. Stable expression of human PSs was monitored by western blotting (WB).

### Western blotting

WB was performed on cell lysates as previously described (Stanga et al., 2016). Primary antibodies used were Anti-OXPHOS Cocktail (1:1,000; Abcam, Cambridge, United Kingdom); Anti-TOM20 (1:1,000; Proteintech, Rosemont, USA); anti-Presenilin (1:1,000; Cell Signaling, Danvers, USA); anti-Actin (1:3,000; Abcam, Cambridge, United Kingdom). Secondary antibodies used were anti-mouse (1:10,000; GE Healthcare, Little Chalfont, United Kingdom) and anti-rabbit (1:10,000; GE Healthcare, Little Chalfont, United Kingdom).

### Metabolic measurements

Oxygen consumption rate (OCR) measurements were performed with a Seahorse XF96 bioenergetic analyzer (Seahorse Bioscience; Massachusetts, USA). Cells were plated at 80% cell confluence onto a Seahorse 96 well plates 24 h before the assay. According to manufacturer's instructions, cell medium was replaced by the conditional medium (culture medium without FBS and sodium bicarbonate) and incubated at 37°C without CO_2_ for 1 h before completion of probe cartridge calibration. Inhibitors targeting the different mitochondrial complexes (Cell Mito Stress Test kit, Seahorse Bioscience) have been added sequentially to the cells during the experiment to measure the basal respiration, the coupling and the spare respiratory capacity: Oligomycin (1 μM); FCCP (1 μM); Rotenone and antimycin A (0.5 μM). Results were normalized to the total amount of protein measured by the Bradford assay kit (Bio-Rad Laboratories, California, USA).

### Mitochondrial membrane potential (Δψ)

Fluorescent cationic probe tetramethylrhodamine methyl ester (TMRM) (Sigma-Aldrich, St Louis, USA) was used to evaluate the ΔΨ. The uncoupling agent Carbonyl cyanide-4-(trifluoromethoxy) phenylhydrazone (FCCP) served as control (Sigma-Aldrich, St Louis, USA). Cyclosporin H (2 μM; Abcam, Cambridge, United Kingdom), an inhibitor of multidrug resistance pump activity was used to limit the efflux of TMRM. MEFs were incubated for 30 min at 37°C with TMRM (30 nM), cyclosporine H with or without FCCP (10 μM) diluted in KREBS medium. For the flow cytometry (FC) analysis we trypsinized cells seeded in 6 well plates and harvested them in PBS/5% FBS. After centrifugation at 300 g for 5 min, cells were resuspended in KREBS medium for the FC analysis performed on a BD FACSCanto™ flow cytometer (Biosciences; San Jose, USA). Data were analyzed with the FlowJo software (FlowJo,LLC; Oregon, USA).

### Adenine nucleotides analysis

24 h after seeding in 10 cm culture dishes, cells were harvested and lysed for 30 min on ice with HCLO_4_ 1N (Sigma-Aldrich, St Louis, USA) and then centrifuged for 20 min at 10,000 g at 4°C. The pellets were re-suspended in NaOH 0.5 M for protein quantification and the supernatants were adjusted to pH 6–8 using a solution of KOH/KHCO_3_ 3 M (Sigma-Aldrich, St Louis, USA). Precipitated salts were separated from the liquid phase by centrifugation at 10,000 g at 4°C for 20 min. Samples were stored at −80°C. Nucleotides were separated by high-performance liquid chromatography (HPLC) on 125 × 4.6-mm PartiSphere 5 SAX anion-exchange column (Whatman, Maidstone, UK). Nucleotides were separated with a gradient from 100% buffer A (0.01 M NH_4_H_2_PO_4_, pH 3.7) to 100% buffer B (0.48 M NH_4_H_2_PO_4_, pH 3.7) over 27 min at a flow rate of 2 ml/min according to the method of Hartwick and Brown (1975). UV detection of nucleotides was performed at 254 nm. Quantification of nucleotides was achieved by peak integration of the area under the curve, validated by the use of external standards (ADP and ATP).

### NAD^+^/NADH ratio

Cells were seeded in 96 well plates at 80% confluence 24 h before the assay. NAD^+^/NADH ratio was measured by using the bioluminescent NAD^+^/NADH-Glo^TM^ assay kit (Promega, Wisconsin, USA) according to the manufacturer's instructions. Briefly, total NAD^+^ and NADH were extracted from cell pellets with the basic solution 1% dodecyltrimethylammonium bromide (DTAB). Samples were divided in two for both acid and basic treatments and heated at 60°C for 15 min. The oxidized form is selectively decomposed in the basic solution while the reduced form is decomposed in the acidic solution. For the luminescent reaction, samples were mixed with 100 μl of NAD^+^/NADH-Glo^TM^ detection reagent and incubated for 45 min before reading on the GloMax® 96-well plate luminometer (Promega, Wisconsin, USA).

### Glucose and lactate measurements

Cells were seeded at 80% confluence in 6 well plates. 24 h after, 500 μl of medium per well was collected and deproteinized. Glucose and lactate concentrations were measured using specific enzymatic assays on a CMA600 microdialysis analyzer (CMA Microdialysis AB, Solna, Sweden). Cells were collected for protein quantification and data were normalized to the amount of protein measured by BCA assay (Thermo Scientific, Rockford, USA).

### Glycolytic flux measurement

Glycolytic rate was evaluated by measurement of the detritiation rate of [3-^3^H] glucose. Briefly, tritiated glucose (0.2 μCi/ml; Perkin-Elmer; Massachussets, USA) was added to the medium (including 5.5 mM glucose) for 30 min. After medium removal, the tritiated water resulting from detritiated glucose was separated from the non-transported tritiated glucose by column chromatography and measured with the Tri Carb 2,810 liquid scintillation analyzer (Perkin Elmer; Massachussets, USA) as described previously (Marsin et al., 2002). Data were normalized to the amount of protein measured by BCA assay (Thermo Scientific, Rockford, USA).

### Complex I (CI) enzyme activity assay

The activity of complex I of the mitochondrial respiratory chain was evaluated with the Complex I Enzyme Activity Dipstick Assay kit (Abcam, Cambridge, United Kingdom). Cells were scraped in PBS and centrifuged for 5 min at 4°C at 500 g. 10 volumes of extraction buffer were added to the pellets prior to 20 min of incubation on ice. After centrifugation, pellets were discarded and supernatants were used to determine protein concentration (BCA assay). Samples (corresponding to 30 μg of proteins) were added to a microplate with blocking buffer. The dipsticks (containing an antibody capturing the CI) were immersed in the samples to capture the CI. The NADH and NBT were added to allow the oxidation of NADH by the complex I, which in turn reduce NBT to form a purple precipitate. Colorimetric signals were quantified with the Gel Doc 2,000 coupled with Quantity One software (Bio-Rad; California, USA).

### Transmission electron microscopy (TEM)

Cells were fixed with 2.5% glutaraldehyde in phosphate buffer and kept in the fixative during 1 h at room temperature. They were washed and postfixed with 1% osmium tetroxide in the same buffer containing 0.8% potassium ferricyanide at 4°C. The samples were dehydrated in acetone, infiltrated with Epon resin during 2 days, embedded in the same resin and polymerised at 60°C during 48 h. Ultrathin sections were obtained using a Leica Ultracut UC6 ultramicrotome (Leica Microsystems, Vienna) and mounted on Formvar-coated copper grids. They were stained with 2% uranyl acetate in water and lead citrate. Sections were observed in a Tecnai Spirit electron microscope equipped with an Eagle CCD camera (FEI, Eindhoven, The Netherlands).

### Statistical analysis

The number of experiments for each experimental condition is indicated in the figure legends. Data were analyzed using GraphPad Prism software (GraphPad Software, La Jolla, CA, USA) by ANOVA followed by Bonferroni's multiple comparison tests. ^*^p < 0.05, ^**^p < 0.01, ^***^p < 0.001.

## Results

### Absence of PS2 results in decreased OXPHOS without alteration of the ΔΨ

**Figure 1:**
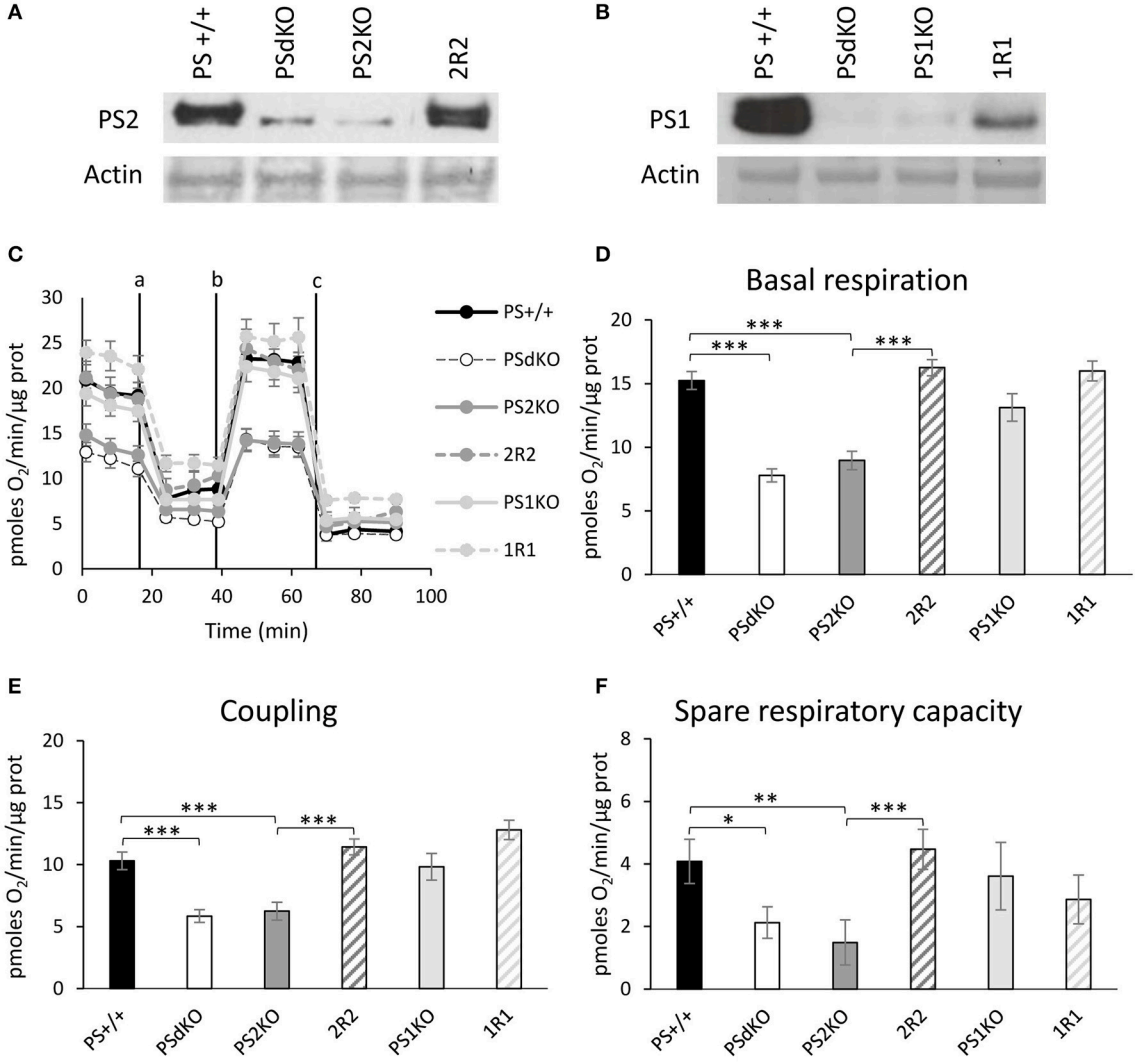
Assessment of the OXPHOS capacity by measurement of the OCR. Experiments were carried out in MEF cell lines wild type (PS+/+), PS1/2 double KO (PSdKO), PS2KO, PS1KO and rescued cell expressing human PS1 and PS2 in PS1KO and PS2KO backgrounds, respectively (2R2 and 1R1). PSs expression **(A,B)** was analyzed by WB in cell lysates. Actin was used as a loading control. **(C)**. OCR was determined using the Seahorse XF96 bioenergetic analyzer. Vertical lines indicate the time point at which the different compounds have been added: a. Oligomycin (CV inhibitor) b. FCCP (ΔΨ uncoupler) c. Rotenone (CI inhibitor) and antimycin A (CIII inhibitor). The basal respiration **(D)**, the coupling ratio **(E)** and the spare respiratory capacity **(F)** were calculated according to the Cell Mito Stress Test kit's recommended protocol. Values (means ± sem) are given in pmol O_2_/min/μg protein.^*^p < 0.05, ^**^p < 0.01, ^***^p < 0.001 (*n* = 18 from 3 independent experiments).

**Figure 2:**
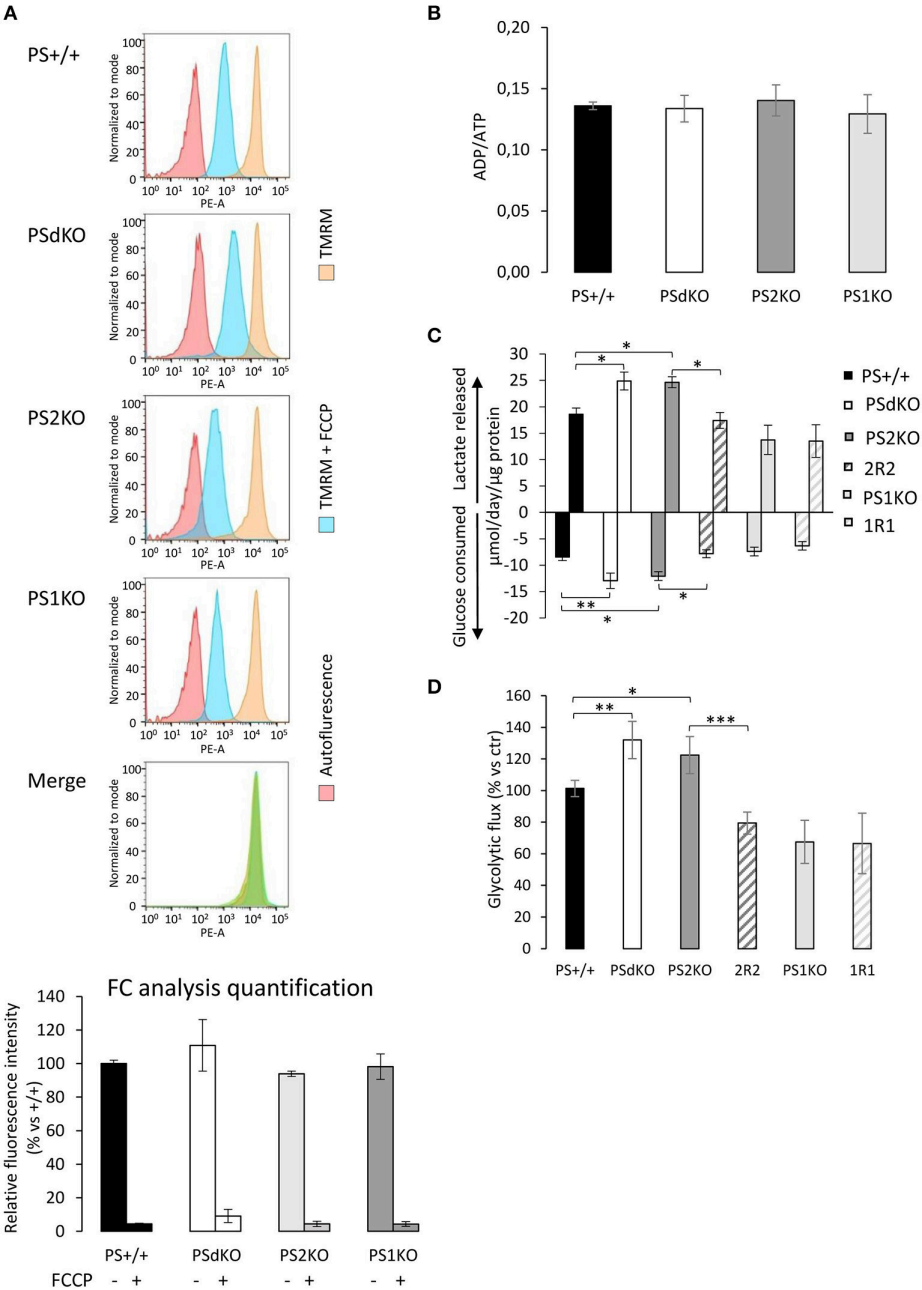
Evaluation of the ΔΨ, ADP/ATP ratio and glycolysis. ΔΨ was evaluated in MEF cells with the TMRM probe in the presence or absence of the ΔΨ uncoupling agent FCCP and analyzed by FC (*n* = 4 from 2 independent experiments) **(A)**. Histogram peaks represent (from left): autofluorescence; relative fluorescence of cells treated with TMRM+FCCP and of cells treated with TMRM only. The merge of histograms (in green) plots the overlapping signal obtained for the 4 cell lines in the TMRM only condition. Signals obtained were quantified (bottom) and results are expressed as the percentage of relative mean fluorescence intensity measured in PS+/+ cells. (**B)**. ADP and ATP were measured by HPLC 24 h after seeding and data, expressed as ADP/ATP ratio, were normalized to protein content (*n* = 6 from 3 independent experiments). **(C)**. Glucose consumption (negative columns) and lactate release (positive columns) were measured in supernatant from fresh medium after 24 h of culture. Data were normalized to protein content and expressed as μmol/day/μg prot. ^*^p < 0.05, ^**^p < 0.01, (*n* = 16 from 6 independent experiments). **(D)**. Glycolysis rate was determined by the detritation rate of [3-^3^H] glucose after a 30 min incubation. Data were normalized to protein content. ^*^p < 0.05, ^**^p < 0.01, ^***^p < 0.001 (*n* = 12 from 6 independent experiments).

PSs expression profile was measured by WB in MEF cell lines with antibodies directed against PS1 and PS2 CTFs (Figures 1A,B). The expression profile indicated the restoration of PSs expression in 2R2 (PS2 KO stably expressing human PS2) and 1R1 (PS1KO stably expressing human PS1). We evaluated the OXPHOS capacity by measurement of the OCR. The overall profile of OCR and the parameters related to the OXPHOS activity measured before or after drug addition, that are basal respiration, coupling (oxygen consumption devoted to ATP synthesis under resting conditions) and spare respiratory capacity (maximal uncoupled rate of respiration minus the basal rate) were all impaired in PSdKO and PS2KO cells (Figures 1C–F). Stable re-expression of PS2 in the PS2KO background (2R2) restored all these parameters to the levels measured in control cells (PS+/+). Importantly, the absence of PS1 did not affect mitochondrial respiration. We next measured the ΔΨ with the TMRM probe by FC (Figure 2A) and by fluorescence measurement in microplates (Figure S1A). FC analyzes were performed on gated homogenous populations that accounted for more than 90% of the total population (Figure S1B). FCCP, an uncoupling agent abolishing ΔΨ was used as a control. In both approaches (FC and direct fluorescence measurements), no significant differences in ΔΨ were observed between the different populations of MEFs, although oxygen consumption was altered in PS2KO and PSdKO cells.

### Increased anaerobic glycolysis sustains the energy production in PSdKO and PS2KO MEFs

The observed PS2-dependent OXPHOS capacity decrease without impairment of ΔΨ led us to evaluate the total ADP and ATP cellular levels. This was achieved by HPLC. No differences were observed in the ADP/ATP ratio between the cell lines (Figure 2B). Considering that we detected a significant decrease in coupling in PSdKO and PS2KO cells, we suspected that ATP production in PSdKO and PS2KO cells could result from an increased glycolysis (Figures 2C,D) that would counteract the defects observed in OXPHOS. We measured the levels of lactate secreted and glucose consumed by cells, as marker of glycolysis (Figure 2C). Both were indeed significantly higher in PSdKO and PS2KO cells. No changes were observed in PS1KO cells. Lactate secreted and glucose consumed measured in 2R2 cells were comparable to those measured in PS+/+ cells. These data were in line with the significant increase of the glycolytic flux (measured by detritiation of [3-^3^H] glucose) observed in PSdKO and PS2KO cells, that was also restored in 2R2 to the levels measured in PS+/+ cells (Figure 2D).

### Absence of PS2 impairs the ETC

**Figure 3:**
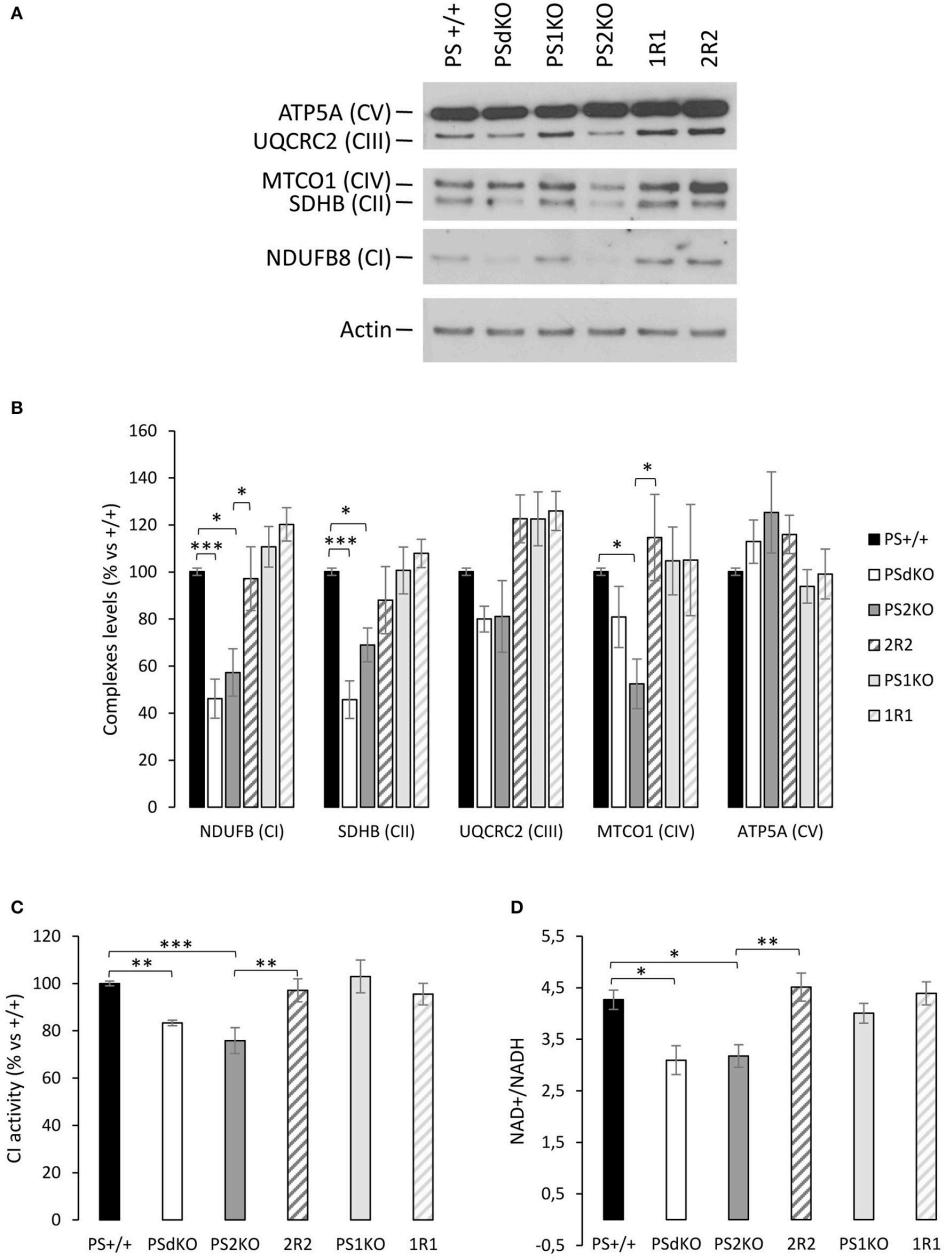
Mitochondrial respiratory chain complexes expression profile, NAD^+^/NADH ratio and CI activity. **(A)**. The expression level of representative protein subunits from each of the five mitochondrial complexes (NDUFB8 for CI; SDHB for CII; UQCRC2 for CIII; MTCO1 for CIV; ATP5A for CV) was analyzed by WB on cell lysates. Actin was used as a loading control (*n* = 5 from 5 independent experiments). **(B)**. WB quantifications (means ± sem) are given as percentage of signal measured in PS+/+ cells.^*^p < 0.05, ^***^p < 0.001. **(C)**. Mitochondrial CI activity was evaluated by the enzyme activity dipstick assay. ^**^p < 0.01, ^***^p < 0.001 (*n* = 12 from 6 independent experiments). **(D)**. NAD^+^/NADH ratio was quantified by a bioluminescent kit ^*^p < 0.05, ^**^p < 0.01 (*n* = 24 from 8 independent experiments).

So far, our results showed that mitochondrial oxidative capacity was impaired in the absence of PS2. This defect can be compensated by the increased glycolytic flux we observed. We further investigated the mitochondrial defects by profiling the expression of the five mitochondrial complexes. This was performed by WB with a cocktail of antibodies targeting representative subunits of the five mitochondrial complexes. Results showed that the expression of some complexes of the ETC was perturbed in PSdKO and PS2KO cells: CI and CII subunits were significantly decreased in both cell lines, and CIV was more specifically decreased in PS2KO cells. The expression profile of CI, CII, and CIV was restored in 2R2 cells (Figures 3A,B). To confirm these observations, we measured by an enzymatic assay the activity of the CI, which was significantly decreased in PSdKO and PS2KO cells but totally restored in 2R2 cells (Figure 3C). Since CI uses NADH as an electron donor, we assessed whether the redox state was also affected by measuring the NAD^+^/NADH ratio. We measured the NAD^+^/NADH ratio using a luminescence assay. Consistent with the measurement of CI subunits by WB, NAD^+^/NADH ratio was decreased in PSdKO and PS2KO cells and restored in 2R2 cells (Figure 3D).

### Mitochondrial morphology in PSs-deficient cells

**Figure 4:**
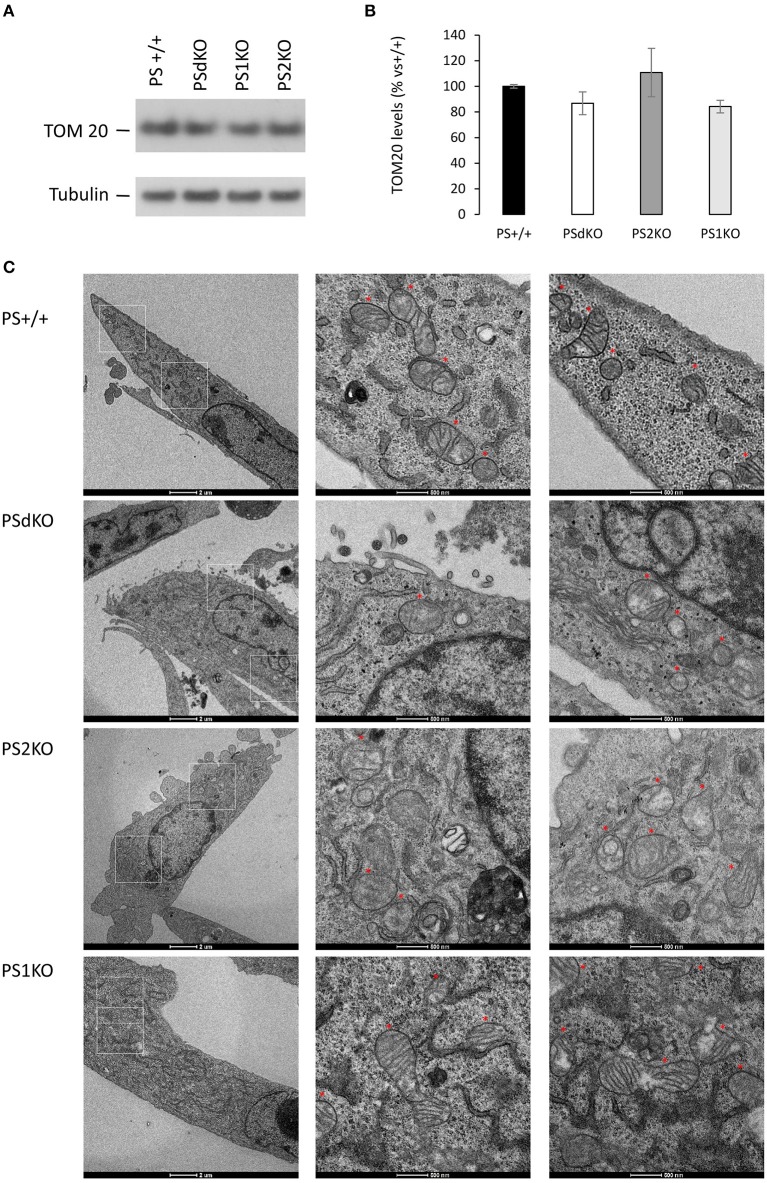
Characterization of mitochondrial mass and morphology. TOM20 expression was analyzed by WB on cell lysates **(A)**. Tubulin was used as a loading control. TOM20 quantification **(B)** (means ± sem) is given as the percentage of the signal measured in the PS+/+ cells (*n* = 6 independent experiments). **(C)** TEM's of cell section. Higher magnification regions (right columns) are boxed in white (left column). Red stars are indicating the position of the mitochondria on the micrograph. Scale bars are indicated on the bottom of the pictures.

We measured by WB in cell lysates the expression level of the mitochondrial import receptor subunit TOM20 (Figures 4A,B) as a marker of mitochondrial mass (Whitaker-Menezes et al., 2011). No significant difference in TOM20 levels was observed between the different cell types suggesting a comparable mass of mitochondria in all the cell lines tested, including PSdKO and PS2KO cells. Mitochondrial mass is a gross indicator that could be only hardly related to the changes in mitochondrial function we observed. Thus, general mitochondrial morphology was evaluated by immunofluorescent staining targeting TOM20 (Figure S2), and no significant changes were observed about the shape and distribution of the mitochondrial network. Morphology and structure of single mitochondria were evaluated by TEM. Strikingly, we observed defective cristae (Figure 4C) in PSdKO and PS2KO cells when compared to PS+/+ cells. Cristae were less defined and less numerous in MEFs PSdKO and PS2KO and, importantly, these morphological defects were not observed in PS1KO cells.

## Discussion

The physiological function of the Presenilins 1 and 2 is far from being fully elucidated. Over the two last decades, the seminal observation identifying PS1 as the catalytic core of the γ-secretase (De Strooper et al., 1998; Kimberly et al., 2003) launched a considerable research effort aimed at understanding the exact role of PSs in γ-secretase activity and Aβ production. However, an increasing number of studies focused on γ-secretase independent PSs functions such as regulation of calcium homeostasis (Zhang et al., 2013) or neurotransmitter (glutamate) release (Zhang et al., 2009). Since PSs were shown to be enriched in MAM, a domain involved in different pathways related to ER and mitochondria functions (van Vliet et al., 2014; Filadi et al., 2017), we investigated the potential role of PSs in the control of cellular metabolism by measuring an array of parameters related to mitochondrial activity in PSs-deficient MEF cell lines. We observed in the absence of PS2, alterations in OXPHOS capacity and integrity associated with a decrease NAD^+^/NADH ratio. Along with these functional defects, the integrity of the mitochondrial cristae was also affected. These defects occurring in absence of PS2 were compensated by an increased glycolysis. Mitochondrial function was fully restored by stable re-expression of PS2 in our model.

### PS2 in OXPHOS and ΔΨ

We measured the OCR as an indirect measurement of the OXPHOS. Our results indicate a significant decrease in PSdKO and PS2KO of the OCR. All the parameters we measured (basal respiration, coupling and spare respiratory capacity) were decreased in PSdKO and PS2KO cells, supporting a general defective OXPHOS capacity. One could have expected here an impact on the ΔΨ and ATP production, since the ATP synthase uses the electrochemical gradient formed by pumping protons through the inner membrane to produce ATP. With the TMRM probe, we observed that the ΔΨ measured is stable in all the cell types. Previous studies reported a ΔΨ decrease in PSdKO and PS2 KO cells (Behbahani et al., 2006). This discrepancy could be due to different procedures as for instance the use of a different probe (JC-1) at high concentration (Perry et al., 2011). Behbahani et al. used like us FC analysis as a readout for ΔΨ, but it should be noted that in their case the use of the JC-1 probe to measure ΔΨ changes implies a shift in the emission wavelength of the probe (with overlapping spectra) that renders the analysis by FC more difficult to interpret. With the TMRM probe, by using FC or direct fluorescent measurement in microplates (see Figure S1) as a readout we observed that the ΔΨ is stable in all the cell types analyzed. ΔΨ is also known to be one of the most stable parameter in the cell; even in the case of a deficit, the ATP synthase will work in reverse to keep it stable (Uechi et al., 2006).

To note, the main source of ROS production is the ETC, and their implication in AD could rise the question about their production in our model. We could expect a decrease of ROS production in PSdKO and PS2KO regarding of the OXPHOS deficit. A previous study showed a ROS increase in PSdKO compared to PS+/+, in serum deprivation conditions (Boo et al., 2009) that we do not modelize here. We performed few pilot ROS measurement (data not shown) in basal state but no significant message came up. It would be of interest, given the implication of ROS in AD, to further study the PSs-dependent and ROS production in relevant models.

### PS2 and ATP production

Surprisingly, the ADP/ATP ratio we measured by HPLC was not impaired while the coupling (oxygen dedicated to ATP production) was significantly decreased in PSdKO and PS2KO cells. This is consistent with the increase of glycolytic flux and lactate production we observed in PSdKO and PS2KO cells. We used two distinct approaches to estimate glycolysis in our model: (i) we measured the levels of glucose consumed and lactate released in cell media; (ii) we measured the glycolysis rate by detritiation of [3-^3^H]glucose. These combined approaches showed that the glycolysis is increased significantly in PSdKO and PS2KO, providing the fuel to compensate their OXPHOS deficit. It should be noted that, similarly to undifferentiated stem cells (de Meester et al., 2014), MEF cell lines use anaerobic glycolysis even in the presence of oxygen to produce their energy. It would be of prime interest to analyze if such compensatory capacity exists in non-proliferative and oxidative cells like primary neurons. The metabolic phenotype resulting from altered PS2 function could be more drastic in neuronal cells. This would be very important in the context of the Alzheimer pathology, in which PSs play a central role. Zampese and collaborators highlighted in SH-SY5Y neuronal cells overexpressing a FAD PS2 mutant an enhanced Ca^2+^ transfer between ER and mitochondria (Zampese et al., 2011b). On the other hand, SH-SY5Y treated with siRNA specific for PS2 showed a decreased contact between mitochondria and ER, which induced a decrease in calcium flux. The calcium crosstalk is known to be an important messenger for Krebs cycle enzymes stimulation and kinetics of the mitochondrial complexes. The mitochondrial calcium sequestration capacity is also important in the excitotoxicity and mitochondrial oxidative stress related to AD. It is therefore possible that a lack of calcium flux could be one cause of the OXPHOS defect observed in our PS2KO cells.

### PS2 in ETC and mitochondrial network and morphology

We found that the bioenergetics defects observed were neither due to a decrease in the mitochondrial mass nor associated with a compensatory induction in mitochondrial mass. This favors the hypothesis of a direct disruption of the ETC activity in absence of PS2 rather than an overall defect in mitochondria biogenesis. This would be interesting to further address in postmitotic cells, such as neurons, where one might expect a proliferation of mitochondria as an attempt to compensate for the OXPHOS deficits. Such a compensatory induction was seen in heart and muscle (and to a lesser extent in brain) of mice deficient in the adenine nucleotide translocator-1 (ANT1), on which OXPHOS depends since it provides the ADP substrate (Esposito et al., 1999)

In order to understand the origin of the OXPHOS defect, we assessed the expression profile of the 5 mitochondrial complexes (CI-CV) of the ETC with a cocktail of antibodies. Even if we did not evaluate the expression of the entire complexes by this approach, we found the expression of several complexes of the ETC (CI, CII, and CIV) to be decreased in PSdKO and PS2KO cells. This was consistent with a functional analysis indicating a decreased CI activity in the same cells. The most important electron donor produced by the Krebs cycle and used by the CI is NADH and NAD^+^ is an important co-factor regulating metabolic homeostasis (Canto et al., 2015). We therefore assessed their redox state by measuring the NAD^+^/NADH ratio and observed a significant decrease of the ratio in PSdKO and PS2KO cells. This can result either from an accumulation of NADH not used by the dysfunctional CI or from a decrease in NAD^+^ associated to metabolic pathways. NAD^+^ and NADH could also both be affected and synergistically influence pathways regulating metabolism homeostasis (Canto et al., 2015).

The perturbation of the integrity of the ETC in PSdKO and PS2KO correlates with an altered structure of their cristae as evidenced by TEM. Cristae are the seat of the ETC, it is thus logical that the OXPHOS capacity and complexes expression are affected when their structure is altered. It has been demonstrated that cristae shape could regulate respiratory chain supercomplexes assembly and stability, impacting the respiratory efficiency (Cogliati et al., 2013). One of the key regulators of cristae shape is OPA1 and it would be interesting to further study the relationship between PS2 and OPA1. Alternatively, considering MAM as an important compartment for the homeostasis of lipids such as for example cardiolipin, one of the most prominent lipids in cristae (Paradies et al., 2014; Ikon and Ryan, 2017), we suggest that the morphological deficit of the cristae could be related to a dysregulation of MAM function in absence of PS2. To note, the MAM hypothesis has been proposed in AD since all the pathways (cholesterol, lipid and calcium homeostasis, mitochondrial OXPHOS stimulation) controlled by this compartment are altered in the pathology (Area-Gomez and Schon, 2017).

In conclusion, we reported here a specific physiological role of PS2 in cellular metabolism. Indeed, absence of PS2 results in defective mitochondrial cristae correlating with an impaired OXPHOS capacity and a modified redox state (NAD^+^/NADH ratio). MEF cell lines, which are glycolytic cells, increase this glycolytic capacity to sustain their energy need in absence of PS2. This implies that impairment of these PS2-dependent processes could be involved in the progression of pathologies like AD in which PS2 play a key role. Investigation in an oxidative model and particularly in neurons are required to further explore this hypothesis.

## Author contributions

SC, SS, and PK-C designed the research study; SC and SS conducted experiments with fundamental input of PEP, MB, and LB; all the authors analyzed data. SC, SS, and PK-C wrote the paper; all the authors have read and approved the final manuscript.

### Conflict of interest statement

The authors declare that the research was conducted in the absence of any commercial or financial relationships that could be construed as a potential conflict of interest.

## Abbreviations

ΔΨ: mitochondrial membrane potential
1R1: PS1 knockout rescued PS1
2R2: PS2 knockout rescued PS2
AD: Alzheimer disease
APP: amyloid precursor protein
CI-CV: complexes I-V
DTAB: dodecyltrimethylammonium bromide
ETC: electron transport chain
ER: endoplasmic reticulum
FAD: familial AD
FC: flow cytometry
FCCP: carbonyl cyanide-4-(trifluoromethoxy) phenylhydrazone
HPLC: high pressure liquid chromatography
MAM: mitochondria-associated membranes
MEF: mouse embryonic fibroblast
OCR: oxygen consumption rate
OXPHOS: oxidative phosphorylation
PS: presenilin
ROS: reactive oxygen species
SAD: sporadic AD
TEM: transmission electron microscopy
TMRM: tetramethylrhodamine methyl ester
WB: western blotting.
