# Supplementary figures and images for: Presenilin 2-Dependent Maintenance of Mitochondrial Oxidative Capacity and Morphology
Source: Front Physiol. 2017 Oct 12;8:796. doi: 10.3389/fphys.2017.00796 (PMC5650731; doi:10.3389/fphys.2017.00796)

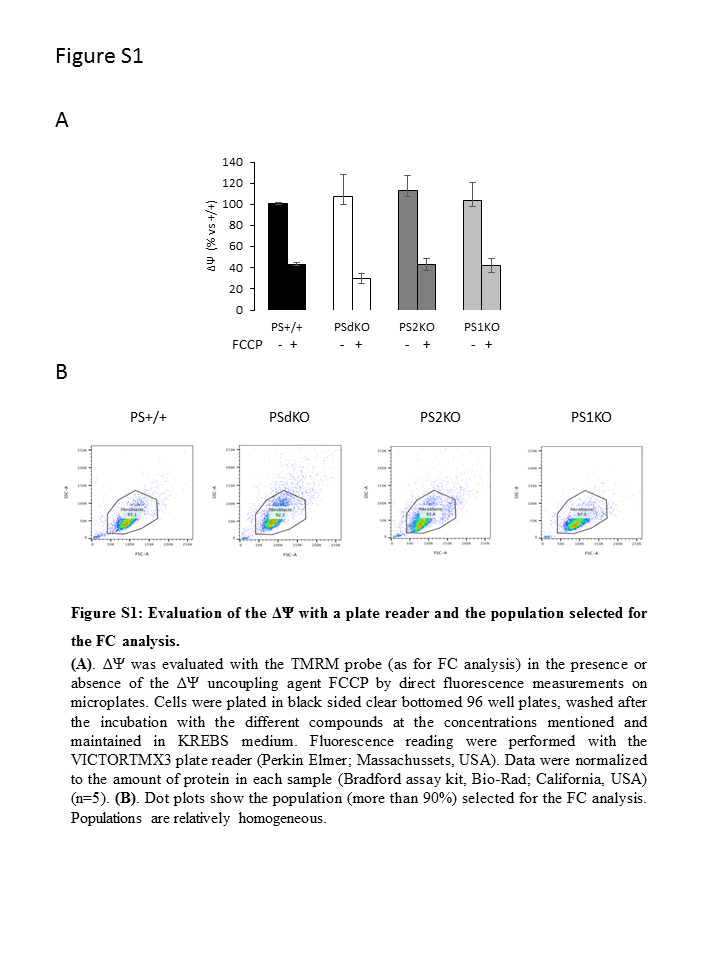

Supplement: Supplementary file 1 [file Image1.tif]

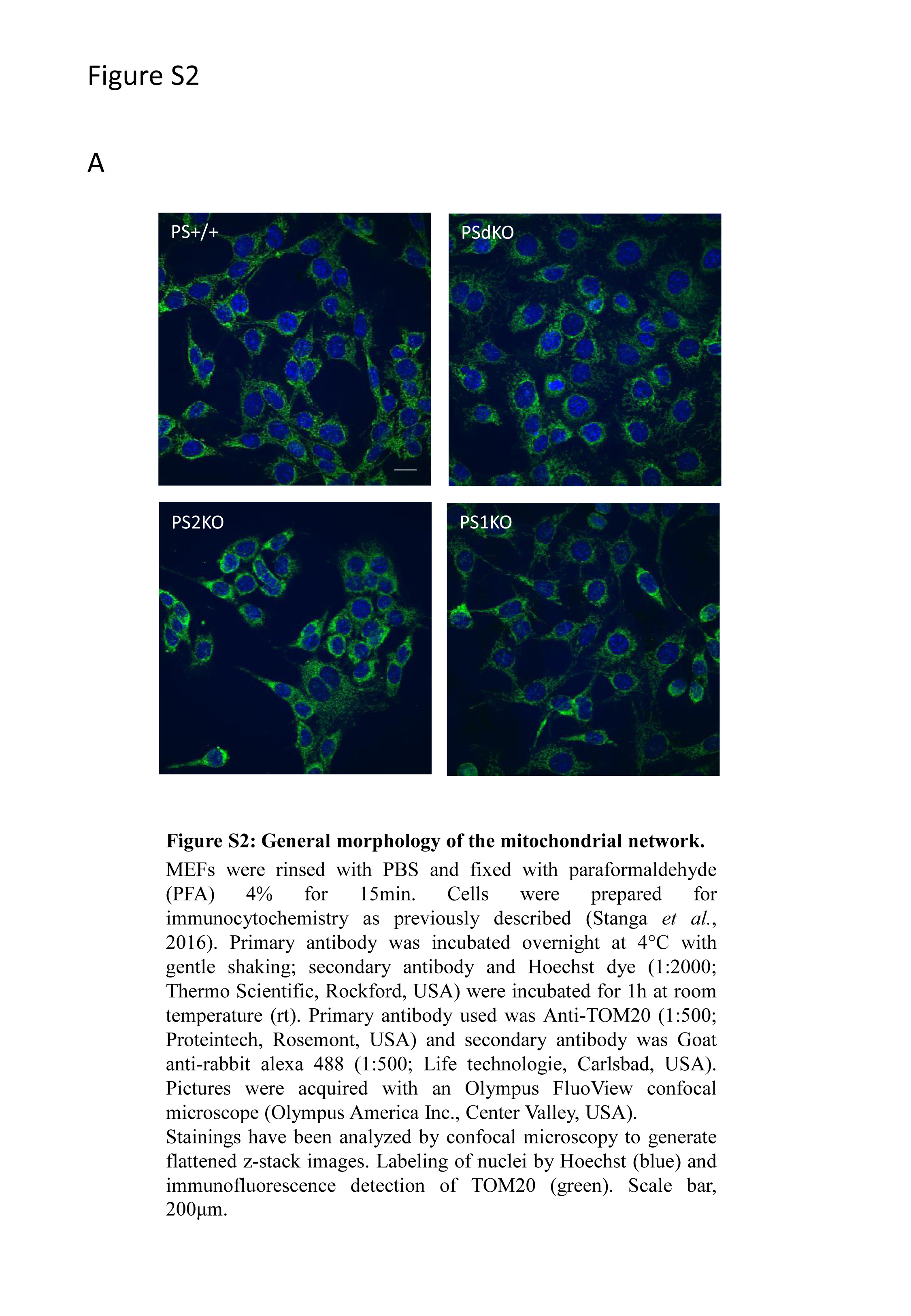

Supplement: Supplementary file 2 [file Image2.JPEG]
